# Characterization of the Mechanical Properties, Water Sorption, and Solubility of Antibacterial Copolymers of Quaternary Ammonium Urethane-Dimethacrylates and Triethylene Glycol Dimethacrylate

**DOI:** 10.3390/ma15165530

**Published:** 2022-08-11

**Authors:** Marta W. Chrószcz-Porębska, Izabela M. Barszczewska-Rybarek, Grzegorz Chladek

**Affiliations:** 1 Department of Physical Chemistry and Technology of Polymers, Faculty of Chemistry, Silesian University of Technology, Strzody 9 Str., 44-100 Gliwice, Poland; 2 Department of Engineering Materials and Biomaterials, Faculty of Mechanical Engineering, Silesian University of Technology, 18a Konarskiego Str., 41-100 Gliwice, Poland

**Keywords:** dimethacrylate copolymers, quaternary ammonium methacrylates, urethane-dimethacrylates, photocurable copolymers, mechanical properties, water behavior

## Abstract

The use of dental composites based on dimethacrylates that have quaternary ammonium groups is a promising solution in the field of antibacterial restorative materials. This study aimed to investigate the mechanical properties and behaviors in aqueous environments of a series of six copolymers (QA:TEG) comprising 60 wt.% quaternary ammonium urethane-dimethacrylate (QAUDMA) and 40 wt.% triethylene glycol dimethacrylate (TEGDMA); these copolymers are analogous to a common dental copolymer (BG:TEG), which comprises 60 wt.% bisphenol A glycerolate dimethacrylate (Bis-GMA) and 40 wt.% TEGDMA. Hardness (*HB*), flexural strength (*FS*), flexural modulus (*E*), water sorption (*WS*), and water solubility (*SL*) were assessed for this purpose. The pilot study of these copolymers showed that they have high antibacterial activity and good physicochemical properties. This paper revealed that QA:TEGs cannot replace BG:TEG due to their insufficient mechanical properties and poor behavior in water. However, the results can help to explain how QAUDMA-based materials work, and how their composition should be manipulated to produce the best performance. It was found that the longer the *N*-alkyl chain, the lower the *HB*, *WS*, and *SL*. The *FS* and *E* increased with the lengthening of the *N-*alkyl chain from eight to ten carbon atoms. Its further extension, to eighteen carbon atoms, caused a decrease in those parameters.

## 1. Introduction

In recent years, a significant increase in the intensity of research into the development of dental restorative materials with antibacterial properties has been observed [1,2]. This results from the fact that teeth and periodontal diseases have become a global problem in the 21st century, and new steps must be taken to keep this issue under control [3,4,5]. Currently, the most commonly used dental restorative materials are dimethacrylate composites that consist of bisphenol A glycerolate dimethacrylate (Bis-GMA) and its derivatives: urethane-dimethacrylate monomer (UDMA), and triethylene glycol dimethacrylate (TEGDMA) [6]. Their popularity is due to their excellent functional properties, esthetics, and low price. However, they do not protect against secondary caries and dental inflammations, because they have negligible antibacterial activity [7,8]. Their modification with bioactive compounds is perhaps the most reasonable means of giving them antibacterial properties. This can be performed in two ways. The first approach uses the admixing of particles of inorganic or organic compounds that have antibacterial effects [9,10]. The main advantage of this approach is its low price. However, such free particles can leach from the composite, as no covalent bonds exist between them and the matrix. This results in a shorter restoration lifetime and increases composite cytotoxicity. The second approach uses the copolymerization of common dimethacrylates with methacrylate monomers containing quaternary ammonium (QA) groups (QAMs) [11,12,13]. As these quaternary ammonium groups are positively charged, they interact with negatively charged bacteria cell walls. This causes an electric imbalance inside the bacteria cell, the leaching of cytoplasmic components that are essential for its proper functioning, an increase in the osmotic pressure inside the cell, and, finally, cell lysis, when the existing risk of cytotoxicity for mammalian cell lines is intracellular in origin. Abnormalities in or damage to intracellular biochemical processes, such as intracellular oxidative stress, oxidative DNA damage, and the induction of intrinsic mitochondrial apoptosis, are involved in lieu of membrane disintegration or cell lysis [14]. Covalent bonding between QAM and other dimethacrylates results in better and more stable physicochemical, mechanical, and biocidal properties of chemically modified composites, compared to physically modified composites [15].

First, monomethacrylates with quaternary ammonium groups (mono-QAMs) were produced [16,17,18,19,20,21,22]. Composites enriched with their presence showed high antibacterial activity against many strains of bacteria, including *Streptococcus mutans*, *Actinomyces viscosus*, and *Lactobacillus casei* [17,18,19,20,21,22]. However, it was found that the mono-QAM-repeating units reduced crosslink density in the composite matrix, which caused the deterioration of its mechanical properties, and led to an increase in water sorption and solubility [19,20,23].

Then, dimethacrylates with quaternary ammonium groups (di-QAM) were developed [23,24,25,26,27,28,29,30,31,32,33,34,35]. They did not decrease copolymer crosslink density, because they have two methacrylate groups [23]. A series of composite materials enriched with their presence showed high antibacterial activity against many strains of bacteria, such as *S. mutans* [23,24,25,28,29,30,32,35], *A. viscosus* [24], *Escherichia coli* [25,27], *Lactobacillus acidophilus* [24], *Streptococcus sanguinis* [24], *Porphyromonas gingivalis* [24], *Prevotella melaninogenica* [24], *Enterococcus faecalis* [24], *Pseudomonas aeruginosa* [25], *Staphylococcus aureus* [24,25,27], and *Bacillus subtillis* [25]. Additionally, the results of these studies showed that the antibacterial activity of composites modified with di-QAMs depended on the number of QA groups in the di-QAM molecule. The more QA groups, the higher the antibacterial activity. Therefore, di-QAM monomers with two QA groups may offer a more promising alternative to those with one QA group, because they achieve an adequate antibacterial effect in the composite at a lower concentration [35]. However, the mechanical and physicochemical properties of copolymers or composites modified with di-QAMs have rarely been examined.

**Figure 1:**
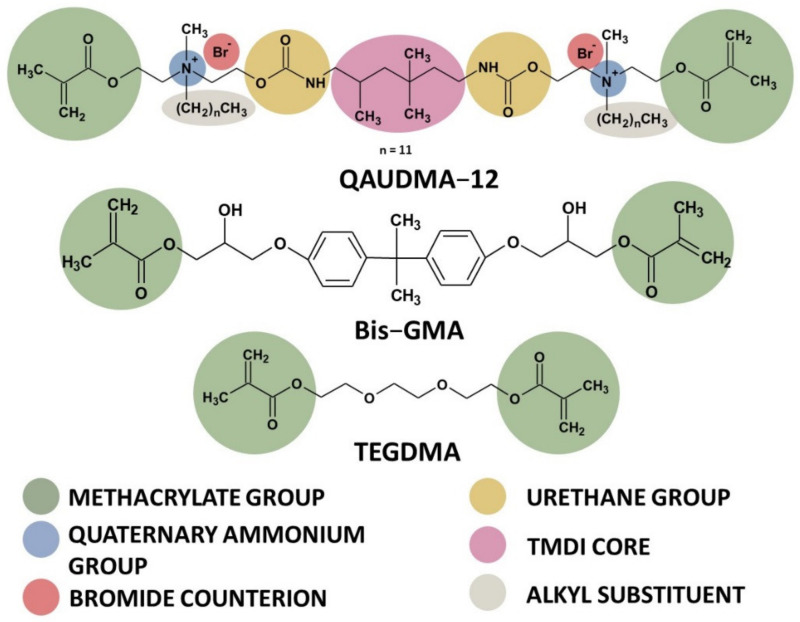
The chemical structure of the QAUDMA, Bis-GMA, and TEGDMA monomers used in this study.

In our previous study [26], we described the synthesis of six novel quaternary ammonium urethane-dimethacrylates (QAUDMAs): namely, the UDMA analogues. They consisted of the trimethylhexamethylene diisocyanate (TMDI) core and two wings. Each wing was terminated with the methacrylate group and contained one quaternary ammonium group substituted with the N-alkyl chain of eight (C8), ten (C10), twelve (C12), fourteen (C14), sixteen (C16), and eighteen (C18) carbon atoms (Figure 1). Novel QAUDMAs show an adequate refractive index, glass transition temperature, and density; however, due to their high viscosity, it is necessary to use a reactive diluent with these monomers for dental applications. Therefore, in next stage, a pilot study on the characterization of the QAUDMA-based polymers was performed for copolymers comprising 60 wt.% QAUDMA and 40 wt.% TEGDMA (Figure 1) (QA:TEGs) [27]. The results of that study showed that QA:TEGs were characterized by a high degree of conversion (*DC*), a high glass transition temperature (*T_gp_*), and low polymerization shrinkage (*S*); they also showed high antibacterial activity against *S. aureus* and *E. coli*, which justified the next phase of the research. The goal of the current investigation was to enhance our knowledge of the properties of QA:TEGs, focusing on their mechanical properties and behavior in the aqueous environment. To this end, hardness, flexural strength, the flexural modulus, water sorption, and water solubility were determined for six QA:TEGs formulations. This type of research is not widely available for QAM-based polymeric materials. The results of this study provide conclusions about the influence of the QAUDMA chemical structure, and in particular the length of the N-alkyl nitrogen substituent, on the physical and mechanical characteristics of their copolymers. Such knowledge has not previously been attained, and it is essential for understanding how a dimethacrylate copolymer containing QAUDMA repeating units works, and how it should be designed to result in the best performance.

## 2. Materials and Methods

### 2.1. Chemicals and Reagents

Alkyl bromides, N-methyldiethanolamine (MDEA), and methyl methacrylate (MMA) were purchased from Acros Organics (Geel, Belgium). Bisphenol A glycerolate dimethacrylate (Bis-GMA), camphorquinone (CQ), dibutyltin dilaurate (DBTDL), 2-dimethylaminoethyl methacrylate (DMAEMA), phenothiazine (PTZ), triethylene glycol dimethacrylate (TEGDMA), and tetramethylsilane (TMS) were purchased from Sigma-Aldrich (St. Louis, MO, USA). Chloroform, methylene chloride, potassium carbonate, and toluene were purchased from POCH S.A. (Gliwice, Poland). Trimethylhexamethylene diisocyanate (TMDI) was purchased from Tokyo Chemical Industry (Tokyo, Japan). All reagents were used as received.

### 2.2. Monomer Synthesis

QAUDMAs were synthesized in a three-stage process as described in our previous work [26]. First, MMA was transesterificated with MDEA in the presence of a reaction catalyst (K_2_CO_3_), a polymerization inhibitor (PTZ), and toluene. The product was isolated from the reaction mixture by washing it with distilled water and chloroform. The crude product was vacuum distilled. It was then N-alkylated with alkyl bromides with alkyl chains of 8, 10, 12, 14, 16, and 18 carbon atoms. The quaternized products were subjected to addition with TMDI in the presence of the reaction catalyst (DBTDL), the polymerization inhibitor (PTZ), and methylene chloride. QAUDMAs were isolated from the reaction mixture by evaporating the solvent. The NMR and FT-IR spectra of QAUDMAs are presented in [26].

### 2.3. Photopolymerization

**Figure 2:**
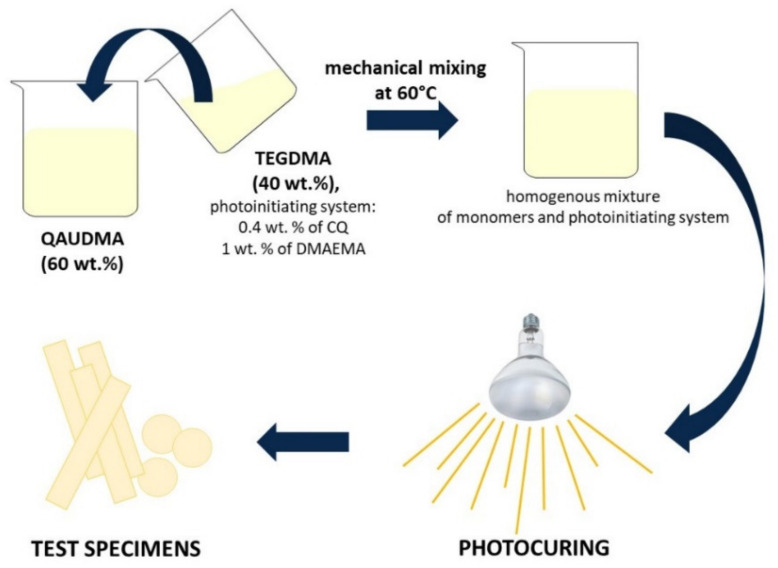
Sample preparation.

Six 60 wt.% QAUDMA and 40 wt.% TEGDMA compositions, and one 60 wt.% Bis-GMA and 40 wt.% TEGDMA composition serving as a reference sample (Table 1), were photopolymerized in the presence of the 0.4 wt.% CQ and 1 wt.% DMAEMA initiating system. Polymerizations were performed in square-shaped glass molds with dimensions of 90 mm × 90 mm × 4 mm (length × width × thickness), and disc-like Teflon molds with dimensions of 15 mm × 1.5 mm (diameter × thickness). A UV-VIS lamp with a 280–780 nm wavelength and 2400 mW/cm^2^ radiation exitance (Ultra Vitalux 300, Osram, Munich, Germany) was used for irradiation. This curing procedure is described in detail in our previous work [27]. The resulting casts were cut into specimens of dimensions dictated by particular standards, and sanded clean with fine sanding paper (Figure 2).

### 2.4. Mechanical Properties

#### 2.4.1. Hardness

Disc-like samples of 40 mm × 4 mm (diameter × thickness) were tested for hardness (*HB*) using VEB Werkstoffprűfmaschinen apparatus (Leipzig, Germany), according to the ISO 2039-1 standard [36].

*HB* was calculated according to the following formula:

(1) $$HB\;\left(MPa\right)=\frac{F_{m}\frac{0.21}{\left(h-h_{r}\right)+0.21}}{\pi dh_{r}},$$

where:

*F_m_*—the test load;

*d*—the diameter of the ball intender (*d* = 5 mm);

*h*—the immersion depth;

*h_r_*—the reduced depth of immersion (*h_r_* = 0.25 mm).

#### 2.4.2. Flexural Properties

Bars of 80 mm × 10 mm × 4 mm (length × width × thickness) were tested for flexural strength (*FS*) and flexural modulus (*E*) using a universal testing machine (Zwick Z020, Ulm, Germany), according to ISO 178 standards [37].

*FS* and *E* were calculated according to the following formulas:

(2) $$FS\;\left(MPa\right)=\frac{3Pl}{2bd^{2}},$$

(3) $$E\;\left(MPa\right)=\frac{P_{1}l^{3}}{4bd^{3}\delta },$$

where:

P_1_—the load at the selected point of the elastic region of the stress-strain plot;

*P*—the maximum load;

*l*—the distance between supports (*l* = 64 mm);

*b*—the sample width (*b* = 10 mm);

*d*—the sample thickness (*h* = 4 mm);

*δ*—the deflection of the sample at *P*_1_.

### 2.5. Water Sorption and Solubility

Disc-like samples of 15 mm × 1.5 mm (diameter × thickness) were tested for water sorption (*WS*) and solubility (*SL*) according to ISO 4049 standards [38].

Samples dried to a constant weight (*m*_0_) were stored in distilled water for seven days at room temperature. After that, the specimens were removed from the water, blotted dry, and weighed (*m*_1_). The samples were then dried again to a constant weight (*m*_2_). Dryings were performed at 100 °C in a conditioning oven. All weightings were performed with an analytical balance (XP Balance, Mettler Toledo, Greifensee, Switzerland) of 0.0001 g accuracy.

*WS* and *SL* were calculated according to the following formulas:

(4) WS μgmm3=m1−m0V,

(5) SL μgmm3=m0−m2V,

where:

m_0_—the initial mass of the dried samples;

*m*_1_—the mass of the swollen samples;

*m*_2_—the mass of the dried samples after immersion in water;

*V*—the initial volume of the dried samples.

### 2.6. Statistical Analysis

The results were expressed as an average value and a corresponding standard deviation (*SD*) achieved for five specimens in each testing method. A non-parametric Wilcoxon test (*p* = 0.05) was used to determine the statistical significance of the results. The calculations were performed using Statistica 13.1 (TIBCO Software Inc., Palo Alto, CA, USA) software.

## 3. Results

A series of six copolymers consisting of 60 wt.% QAUDMA and 40 wt.% TEGDMA (QA:TEG) was subject to an investigation that included the measurement of mechanical properties and behavior in water. The copolymer of 60 wt.% Bis-GMA and 40 wt.% TEGDMA served as a reference sample (BG:TEG). The sample names and their compositions are specified in Table 1.

### 3.1. Mechanical Properties

The summarized results of the mechanical properties *HB*, *FS*, and *E* are given in Table 2.

The *HB* values of the QA:TEGs ranged from 51.41 to 42.17 MPa. They decreased as the length of the N-alkyl substituent increased. All of the QA:TEGs were characterized by lower *HB* values than the BG:TEG reference sample (*HB* = 107.56 MPa). All of these differences were statistically significant. The highest *HB* value was found for the QA8:TEG, which was lower by 52% in comparison to the BG:TEG reference sample. The differences between QA8:TEG, QA10:TEG, and QA12:TEG were statistically insignificant, as were those between QA14:TEG, QA16:TEG, and QA18:TEG. However, the *HB* values of the copolymers of the first group were statistically significantly higher, compared to the *HB* values of the copolymers of the latter group.

The *FS* values of the QA:TEGs ranged from 37.37 to 20.13 MPa. All of the QA:TEGs were characterized by lower *FS* values than the BG:TEG reference sample (*FS* = 51.63 MPa). All of these differences were statistically significant. The highest *FS* was found for the QA10:TEG (*FS* = 37.37 MPa), which was 28% lower than the BG:TEG reference sample. The second highest *FS* value was found for the QA12:TEG (*FS* = 34.46 MPa). In comparison to QA8:TEG (*FS* = 21.59 MPa), the *FS* values of QA10:TEG and QA12:TEG were statistically significantly higher, by 73 and 60%, respectively. They were also statistically significantly higher compared to the *FS* values of the remaining QA:TEGs. QA16:TEG was characterized by the lowest *FS* value (*FS* = 20.13 MPa). In comparison to QA8:TEG, this value was lower by 6%. This value was also slightly lower than the *FS* value of the QA18:TEG (*FS* = 21.75 MPa). These differences were statistically insignificant.

The *E* values of the QA:TEGs ranged from 459.4 to 851.6 MPa. All of the QA:TEGs were characterized by lower *E* values than the BG:TEG reference sample (*E* = 2800.9 MPa). All of these differences were statistically significant. The highest *E* was found for the QA10:TEG (*E* = 851.6 MPa), which was 70% lower than the BG:TEG reference sample. The second highest *E* was found for QA12:TEG (*E* = 848.9 MPa). In comparison to the QA8:TEG (*E* = 679.0 MPa), the *E* values for QA10:TEG and QA12:TEG were statistically significantly higher by 25%. They were also statistically significantly higher than the *E* values of the remaining QA:TEGs. QA18:TEG was characterized by the lowest *E* value (*E* = 459.4 MPa). In comparison to QA8:TEG, this value was lower by 32%. This difference was statistically significant.

### 3.2. Water Sorption and Solubility

**Figure 3:**
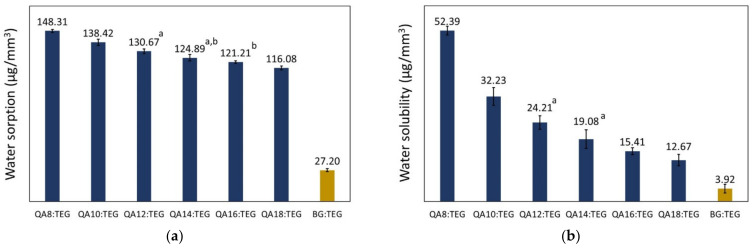
Water sorption (**a**) and water solubility (**b**) of the studied copolymers. Lower case letters indicate statistically insignificant differences (*p* > 0.05, non-parametric Wilcoxon test).

The summarized results related to water sorption (*WS*) and water solubility (*SL*) are given in Figure 3.

The *WS* values of the QA:TEGs ranged from 116.08 to 148.31 µg/mm^3^. All of the QA:TEGs were characterized by higher *WS* values than the BG:TEG reference sample (*WS* = 27.20 µg/mm^3^). The *WS* values decreased as the length of the N-alkyl substituent increased. In addition, all of these decreases were statistically significant. The lowest *WS* value was found for QA18:TEG, which was higher by 326% in comparison to the BG:TEG reference sample.

The *SL* values of the QA:TEGs ranged from 12.67 to 52.39 µg/mm^3^. All of the QA:TEGs were characterized by higher *SL* values than the BG:TEG reference sample (*SL* = 3.92 µg/mm^3^). The *SL* values decreased as the length of the N-alkyl substituent increased. In addition, all of these decreases were statistically significant. The lowest *SL* value was found for QA18:TEG, which was 223% higher than that of the BG:TEG reference sample.

## 4. Discussion

Dimethacrylate monomers containing quaternary ammonium groups have been recognized as compounds with high antibacterial activity. Therefore, using them to produce novel dental composite matrices represents a potential solution for caries treatment. In recent years, many studies of the development of new methacrylate structures containing quaternary ammonium groups have been conducted. However, the focus of these studies is often limited to the determination of the antimicrobial activity of the polymers.

This work is a continuation of research into the new urethane-dimethacrylate monomers with quaternary ammonium groups and their copolymers. The results of previous works revealed the promising physicochemical characteristics of QAUDMA monomers [26], as well as the structural, physicochemical, and antibacterial properties of their copolymers with TEGDMA [27]. This work was intended to enhance our knowledge of the influence of QAUDMAs on the mechanical properties and behaviors in water of their copolymers with TEGDMA.

Matrices in dental composite restorative materials often consist of 60 wt.% Bis-GMA and 40 wt.% TEGDMA, where Bis-GMA is responsible for high mechanical performance, and TEGDMA acts as a reactive diluent. Therefore, this work aimed to verify how the complete replacement of Bis-GMA with the new QAUDMAs would affect the copolymers’ mechanical properties and behavior in water.

### 4.1. Mechanical Properties

Mechanical properties are responsible for dental materials’ performance and capabilities under particular stresses present in the oral environment. These mechanical properties should be determined to assess the proper functioning and usefulness of dental materials, as well as to evaluate the limitations that result from composition and/or curing parameters (initiation systems, irradiation sources, etc.) [39]. Common mechanical properties that are usually considered are hardness, stiffness, and strength.

#### 4.1.1. Hardness

Hardness (*HB*) is defined as the resistance to permanent surface indentation. Adequate *HB* provides dental restoration materials with suitable resistance to stresses arising from mastication and abrasion [40].

The tested QA:TEGs were characterized by *HB* values lower than that of the BG:TEG reference sample. Such a result suggests that studied QA:TEGs are characterized by insufficient *HB* values; therefore, they cannot be used as matrices in dental composites. The results for *HB* can be interpreted from a structural perspective. The decrease in *HB* values that accompanied the increase in the length of the N-alkyl substituent did not show a correlation with the *DC*, which is a structural parameter that strongly influences poly(dimethacrylate)s’ hardness [41]. The QA:TEGs were characterized for *DC* in our previous study [27] and those results are shown in Table 1. As can be seen, the QA:TEGs had high *DC* values, which ranged from 84.0 to 88.7%, and the length of the N-alkyl substituent had no visible influence. These *DC* values can be classified as high, as the *DC* value of the BG:TEG reference sample was 64.8%. As the *DC* did not influence the *HB*, the length of the N-alkyl substituent is probably the key factor affecting *HB*. The precise analysis of hardness showed that QA8:TEG, QA10:TEG, and QA12:TEG had similar *HB* values. A similar situation was observed for QA14:TEG, QA16:TEG, and QA18:TEG. However, the *HB* values of the first group were higher than the *HB* values of the latter group. The lengthening of the N-alkyl substituent from C8 to C12 did not cause a significant decrease in the *HB* values. However, a further increase in its length caused a notable decrease in the *HB* values.

#### 4.1.2. Flexural Strength

Flexural strength (*FS*) is a key factor related to the durability of dental restorative materials. Its value represents the pressure that the material can withstand before breaking. The higher the *FS* value, the higher the stress that the material can withstand [42].

**Figure 4:**
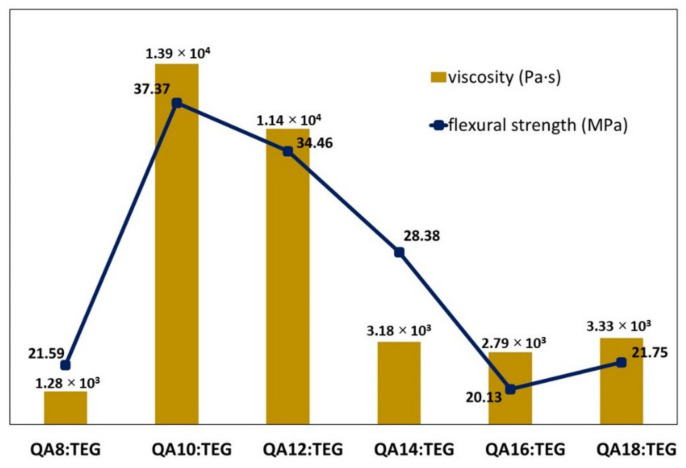
Flexural strength of the QA:TEG copolymers and viscosity of the QAUDMA monomers.

The tested QA:TEGs were characterized by *FS* values lower than that of the BG:TEG reference sample. The initial lengthening of the N-alkyl substituent from C8 to C10 caused a significant increase in the *FS* value. Its further lengthening caused a decrease in *FS* values. This trend did not have any correlation with the *DC* values, which were high for the studied QA:TEGs. The trend of the *FS* values can be explained by the strength of intermolecular interactions between the QAUDMA repeating units in the QA:TEG. This hypothesis can be justified by a comparison of the *FS* values determined for QA:TEGs with the viscosity values of QAUDMAs, which were determined in our previous work (Figure 4) [26].

Viscosity is a common indicator of intermolecular interactions present between monomer molecules. The higher the strength of the molecular interactions, the higher the viscosity, and the more limited the molecular movement [43]. As can be seen in Figure 4, the viscosity of the QAUDMA monomers initially increased with the increase in the length of the N-alkyl substituent from C8 to C10, and its maximum was observed for QA10:TEG. Further lengthening resulted in a decrease in viscosity values. This confirms the hypothesis about the dependency of *FS* on viscosity.

#### 4.1.3. Flexural Modulus

The flexural modulus (*E*) is a key factor that refers to the stiffness of dental restorative materials. Its value represents the ratio between bending stress and the strain measured in the linear elastic region of a material.

**Figure 5:**
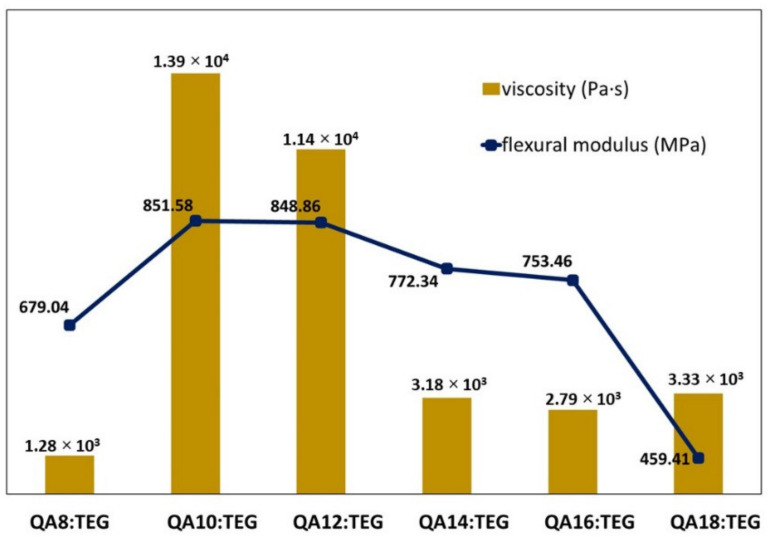
Flexural modulus of the QA:TEG copolymers and viscosity of the QAUDMA monomers.

The tested QA:TEGs were characterized by *E* values lower than that of the BG:TEG reference sample. The initial lengthening of the N-alkyl substituent from C8 to C10 caused a significant increase in the *E* value. Its further lengthening caused a decrease in *E* values. As in the case of *FS*, the trend observed for the *E* values did not correlate with the *DC* values, and is related to the strength of the intermolecular interactions between QAUDMA units (Figure 5).

### 4.2. Water Sorption and Solubility

Water sorption (*WS*) and solubility (*SL*) are two physicochemical factors crucial for the proper functioning of dental restorative materials.

#### 4.2.1. Water Sorption

Excess water absorbed by the dental restorative material usually deteriorates its mechanical properties and has a plasticizing effect on the matrix by decreasing its glass transition temperature [44]. It can also lead to volumetric expansion, resulting in tooth or restoration breakdown [45]. Therefore, the *WS* of dental materials should be assessed; according to the ISO 4049 standard [38], its value cannot be greater than 40 µg/mm^3^. The tested QA:TEGs were characterized by *WS* values greater than that given in the ISO standard, as well as that of the BG:TEG reference sample. The percentage differences over the value indicated in the ISO standard increased as the length of the N-alkyl chain decreased, and ranged from 192 to 271%. Therefore, none of the six QA:TEGs could be applied as a matrix in dental restorative materials.

The results for *WS* can be explained with reference to monomer chemical structures and copolymer crosslink density.

Two quaternary ammonium groups in the QAUDMA molecule are probably the main factor responsible for the high *WS*. This is due to the presence of both positively and negatively charged ions that are prone to absorb water [31].

**Figure 6:**
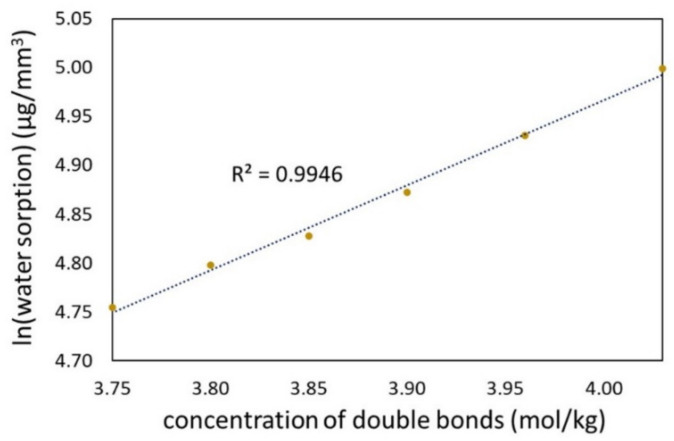
The correlation between the water sorption of the QA:TEG copolymers and the concentration of double bonds in the corresponding monomer compositions. The dotted line - the trendline, the yellow points show the mean values of water sorption.

The QA:TEGs are characterized by lower crosslink density compared to the BG:TEG reference sample. The concentration of methacrylate double bonds in the monomer mixture was used as a parameter to assess the chemical crosslink density in the corresponding copolymer. As can be seen from Table 1, those values decreased as the length of the N-alkyl substituent increased, which means that crosslink density decreases according to the same order. A more detailed analysis of the *WS* values showed that they had a high linear correlation with the concentration of double bonds on a semi-logarithmic scale (Figure 6).

The relationship between the *WS* values and the concentration of double bonds is very interesting, as the latter parameter is only diversified by the molecular weight of the N-alkyl chain in QAUDMA. It might be suspected that the increase in the length of the quaternary nitrogen substituent would cause the loosening of the copolymer network structure. Consequently, water would be able to migrate into it more easily, causing an increase in the *WS*. However, we observed the opposite effect. This can be explained by the following factors. First, the previous study on QA:TEGs shows that the N-alkyl chain takes up less space than suspected, probably due to a coiled conformation [27]. Second, as the length of the N-alkyl chain increases, the quaternary nitrogen structural region gains hydrophobicity. This hypothesis is confirmed by the results achieved in the previous work pertaining to the water contact angle of QA:TEGs. It was found that the hydrophobicity of the QA:TEGs’ surface, quantified by the water contact angle values, increased with the increase in the length of the N-alkyl substituent [27]. Third, there is also a hypothesis in the literature that long N-alkyl chains can adopt specific conformations that obscure the quaternary nitrogen atoms, reducing water affinity [46].

QA:TEGs are also physically crosslinked due to the formation of hydrogen bonds with the hydrogen donor of the QAUDMA urethane linkages. Such hydrogen bonds could cause a slight decrease in *WS* values. Comparing the *WS* values of the BG:TEG reference sample to the other common dental composition containing the urethane-dimethacrylate monomer (UDMA) (40 wt.% Bis-GMA, 40 wt.% UDMA, 20 wt.% TEGDMA, *WS* = 25.64 µg/mm^3^ [47]), the latter’s *WS* value is 6% lower. The fact that QA:TEGs have much higher *WS* values than the BG:TEG reference sample indicates that physical crosslinks involving urethane hydrogen bonds were insufficient to reduce water absorption.

#### 4.2.2. Water Solubility

Water solubility (*SL*) results from the presence of sol fraction [39]; this consists of low molecular weight structures, including monomer molecules, which are not chemically incorporated into the copolymer network. Leaching of sol fraction has an adverse effect on the proper functioning of dental restorative materials, for the following reasons. First, sol fraction is related to incomplete curing [48,49]. Therefore, the mechanical properties of dental restorations might significantly differ from the theoretical level. Second, the elution of the sol fraction may cause the appearance of voids inside the restoration, which would weaken it mechanically [50,51]. Third, the biocompatibility of the filling is reduced. The sol fraction usually has significant cytotoxicity, and therefore its eluting may have harmful effects on surrounding tissues or have a negative impact on organisms [52]. Therefore, the *SL* of dental materials should be assessed; according to the ISO 4049 standard [38], its value cannot be greater than 7.5 µg/mm^3^. The tested QA:TEGs were characterized by *SL* values greater than that given in the ISO standard, as well as that of the BG:TEG reference sample. The percentage differences over the value indicated in the standard increased as the length of the N-alkyl chain decreased, and ranged from 69 to 599%. Therefore, none of the QA:TEGs could be used as a matrix in dental restorative materials. This result can be explained with reference to monomer chemical structures and molecular weights.

**Figure 7:**
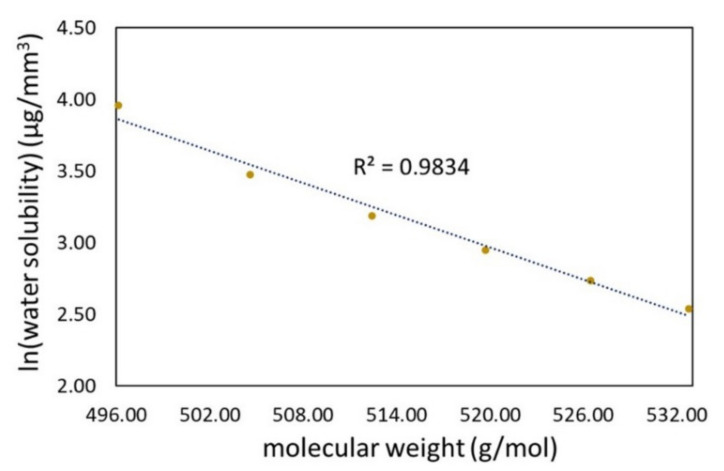
Correlation between the water solubility of the QA:TEG copolymers and the molecular weight of the corresponding monomer compositions. The dotted line-the trendline, the yellow points show the mean values of water solubility.

Since the quaternary ammonium groups have a high affinity to water, the QAUDMA sol fraction can easily migrate from the restoration to an aqueous environment. Detailed analysis of the *SL* values shows that they have a high linear correlation with *MW* on a semi-logarithmic scale (Figure 7).

The *MW* values of the QA:TEG monomer compositions are high (Table 1). The relationship found for the *SL* and *MW* values may indicate that monomers with longer N-alkyl substituents have more difficulty leaching into the aqueous environment, due to the larger size of the monomer molecule. Another factor limiting sol fraction leaching can result from the length of the N-alkyl substituent, which had diverse hydrophobicity. The longer the N-alkyl substituent, the greater the *MW*, and the higher the hydrophobicity [27,53].

## 5. Conclusions

A series of six 60 wt.% QAUDMA and 40 wt.% TEGDMA copolymers were characterized for their mechanical properties and behaviors in water. All of the tested properties depended on the N-alkyl substituent length in the QAUDMA repeating unit. Hardness decreased as the length of the N-alkyl chain increased. Flexural strength and modulus initially increased as the length of the N-alkyl chain increased, up to ten carbon atoms. Its further lengthening caused a decrease in those values. The changes in the flexural strength and modulus were similar to the changes in the viscosity of the QAUDMA monomers, which may be attributed to the changes in the strength of intermolecular interactions between monomer units. Water sorption and solubility decreased as the length of the N-alkyl chain increased. Water sorption revealed a correlation with the concentration of double bonds in the QA:TEG monomer compositions, whereas water solubility revealed a correlation with the molecular weight of QA:TEG monomer compositions.

The values of the mechanical properties, water sorption, and solubility obtained for QA: TEGs indicate that their chemical composition is unsuitable for potential matrices of dental restorative composites. As such, additional investigations into biological properties such as cytotoxicity, or more sophisticated antimicrobial tests, are not justified for these materials. Further research into the QAUDMA-based copolymers must be conducted to obtain materials characterized by adequate values of all physicochemical and mechanical properties. This study provided general insight into the influence of the N-alkyl substituent length on hardness, flexural strength, modulus, water sorption, and solubility, which can help to design QAUDMA-based copolymers of suitable performance.

## Figures and Tables

**Table 1:** Compositions and structural parameters of the studied 60 wt.% QAUDMA and 40 wt.% TEGDMA (QA:TEG) liquid monomer compositions.

Sample Name	Sample Composition			Structural Properties of the Liquid Monomer Compositions		
	QAUDMA		TEGDMA			
	Number of Carbon Atoms in the N-alkyl Chain	Mole Fraction	Mole Fraction	Molecular Weight (g/mol)	Concentration of Double Bonds (mol/kg)	Degree of Conversion ^1^ (%)
QA8:TEG	C8	0.31	0.69	496	4.03	84.2
QA10:TEG	C10	0.30	0.71	505	3.96	84.0
QA12:TEG	C12	0.28	0.72	512	3.90	86.0
QA14:TEG	C14	0.27	0.73	520	3.85	88.7
QA16:TEG	C16	0.26	0.74	526	3.80	87.1
QA18:TEG	C18	0.26	0.74	533	3.75	87.1
	**Bis-GMA**					
BG:TEG	-	0.46	0.54	389	5.14	64.8

^1^ taken from [27].

**Table 2:** Mechanical properties of the studied copolymers: hardness (*HB*), flexural strength (*FS*), and flexural modulus (*E*). Lower case letters indicate statistically insignificant differences (*p* > 0.05) with a column (non-parametric Wilcoxon test).

Sample Name	HB (MPa)		FS (MPa)		E (MPa)	
	Average	SD	Average	SD	Average	SD
QA8:TEG	51.41 ^a,b^	4.32	21.59 ^a,b^	0.66	679.0	36.2
QA10:TEG	51.17 ^a,c^	6.93	37.37 ^c^	2.27	851.6 ^a^	47.4
QA12:TEG	50.87 ^b,c^	4.08	34.46 ^c^	2.18	848.7 ^a^	24.7
QA14:TEG	41.60 ^d,e^	3.63	28.38	1.38	772.3 ^b^	31.1
QA16:TEG	41.21 ^d,f^	2.27	20.13 ^a^	1.62	753.5 ^b^	31.8
QA18:TEG	42.17 ^e,f^	1.08	21.75 ^b^	1.90	459.4	34.4
BG:TEG	107.56	5.70	51.63	6.76	2800.9	78.9

## Data Availability

Data supporting the reported results are available from the authors.
